# Walnut Peptide KG-7 Alleviates Scopolamine-Induced Memory Deficits and Enhances Paracellular Transport via Tight Junction Modulation in a Mouse Model

**DOI:** 10.3390/foods15030548

**Published:** 2026-02-04

**Authors:** Mengqi Li, Junchao Wang, Yutong She, Yuqing Ji, Dan Wu, Yinli Li, Yi Zheng

**Affiliations:** 1 College of Food and Biological Engineering, Xuzhou University of Technology, Xuzhou 221018, China; 2 College of Food Science and Engineering, Jilin Agricultural University, Changchun 130118, China

**Keywords:** tight junctions, scopolamine, hippocampus, bioactive peptides, paracellular absorption

## Abstract

Walnut peptide Lys-Gly-His-Leu-Phe-Pro-Asn (KG-7) is a food-derived bioactive peptide with a high antioxidant capacity. We systematically evaluated the ameliorative effects of KG-7 on scopolamine-induced memory deficits in mice and its intestinal absorption mechanisms through integrating motion behavior analysis, molecular biochemistry research, and fluorescence imaging technology. Morris water maze tests revealed that KG-7 significantly improved the behavioral performance of these mice. Further mechanistic investigations demonstrated that KG-7 restored cholinergic function by reducing acetylcholinesterase activity and increasing acetylcholine levels. Hematoxylin-eosin staining and hippocampal immunohistochemistry confirmed that KG-7 alleviated neuronal damage by downregulating Hes1 overexpression, clarifying its behavioral improvement mechanism. In vitro fluorescence imaging showed that KG-7 reached peak accumulation in brain tissue 8 h post-administration, confirming its brain delivery. To elucidate the absorption mechanism, immunohistochemistry and immunofluorescence revealed that KG-7 markedly reduced the expression of efflux transporter P-gp in the small intestine, thereby diminishing efflux activity, while weakened tight junction (Occludin, ZO-1) fluorescence indicated activation of the paracellular pathway. Western blot analysis confirmed that KG-7 enhanced paracellular absorption efficiency and reduced intestinal efflux by downregulating ZO-1, Occludin, and efflux transporters (P-gp, BCRP, and LRP1) alongside upregulating Claudin-2 expression. These findings provide a foundation for exploring walnut peptides that enhance memory and optimize absorption.

## 1. Introduction

In numerous neurological disorders, prospective memory deficits emerge in the early stages and worsen as the disease progresses [1]. In recent years, the prevalence of memory disorders in the elderly has continued to rise, along with an increase in neurological conditions such as dementia and Alzheimer’s disease (AD) [2]. Memory impairment is one of the main characteristics of neurodegenerative diseases such as AD; however, its pathogenesis is currently underexplored. Neuronal apoptosis, neurotransmitter disorders, neuroinflammatory factors, oxidative stress, autophagy, mitochondrial dysfunction, and Ca^2+^ channels are known to be widely involved in the pathogenic process [3,4,5], and it is therefore evident that the pathogenesis of memory disorders is induced by a variety of pathways. As such, it is necessary to develop functionally active substances that can enhance cognitive ability and reduce memory deficits. Several studies have shown that bioactive peptides derived from food sources have potential for mitigating neurodegeneration and ameliorating memory deficits [6]. In this respect, walnut peptides, which contain essential amino acids, have gained increasing attention as food-derived peptides [7]. According to the study of Li et al., walnut peptide KG-7 completely passes through the small intestine, has excellent antioxidant activity, and can improve memory [8]. In addition, Wang et al. [9] demonstrated that oral supplementation with walnut peptides reduced memory deficits through acetylcholinesterase (AChE) inhibition and upregulation of the antioxidant regulator Nrf2. Furthermore, through histopathological analysis using hematoxylin-eosin (HE) and Nissl staining, Geng et al. [10] found that the walnut peptide WNP-10 reduced hippocampal neuronal damage while enhancing spatial learning and memory retention in scopolamine-induced amnesic mice. These findings collectively suggest that dysregulated hippocampal AChE activity and neuronal apoptosis are key mechanisms underlying memory dysfunction. This study focused on investigating the inhibition of AChE activity and the protection of hippocampal neurons in relation to the administration of KG-7 in a mouse model.

Scopolamine is a nonselective muscarinic receptor antagonist that impairs cholinergic nerve conduction and causes memory impairment, leading to amnesia in animal models, which disrupts the cholinergic system and leads to cognitive decline. Similarly to scopolamine, other nonselective muscarinic receptor antagonists, such as atropine, as well as muscarinic receptor antagonists that selectively target specific subtypes like pirenzepine, trihexyphenidyl, benztropine, biperiden, and dicyclomine, can all lead to learning and memory impairments. Despite the peripheral side-effects of high-dose scopolamine and its non-specific impacts on behavior (e.g., changes in pupil diameter, salivation and other smooth muscle function-related effects), the model remains widely used in research related to memory impairment [11,12].

Although the potential of bioactive peptides has been demonstrated to promote human health, their efficacy remains limited by poor oral bioavailability [13]. The pharmacological activity of bioactive peptides depends on their systemic bioavailability, which is influenced by gastrointestinal stability, barrier permeability, and tissue-specific biodistribution. The small-intestinal epithelium is the principal absorption site for bioactive peptides within the gastrointestinal system. Dietary bioactive peptides enter the systemic circulation through four main transport mechanisms across the intestinal epithelium: carrier-mediated transport, endocytotic absorption, paracellular diffusion, and passive transcellular diffusion [14]. Paracellular absorption, a form of passive diffusion, is the most commonly documented absorption pathway for food-derived bioactive oligopeptides [15]. The paracellular barrier consists of tightly regulated intercellular junction complexes primarily governed by the apical tight junction (TJ). Li et al. [16] reported that the paracellular transport of the shrimp peptide Gln-Met-Asp-Asp-Gln (QMDDQ) is mediated through MLCK-dependent phosphorylation signaling, which induces the structural modulation of TJs in the intestinal epithelium. In addition, Zhang et al. [17] used a Caco-2 monolayer model to demonstrate that the soybean peptide WGAPSL is transported mainly through paracellular pathways via TJs. As such, KG-7 is a low-molecular-weight food-derived oligopeptide and a potential paracellular transport molecule that requires further investigation.

This study was designed to systematically evaluate the efficacy of the walnut peptide KG-7 against scopolamine-induced memory dysfunction in a murine mouse model while also elucidating its intestinal absorption mechanisms. The effects and mechanisms of action of KG-7 against scopolamine-induced memory deficits were evaluated using Morris Water Maze (MWM) tests combined with hippocampal assessments of AChE activity, acetylcholine (Ach) levels, HE staining, and immunohistochemistry. The metabolic rate of KG-7 in brain tissue was examined through fluorescence imaging. Intestinal absorption pathways and the regulation of efflux transport proteins were investigated using immunohistochemistry, immunofluorescence, and western blotting. This study provides a theoretical basis for developing functional foods that support memory improvement.

## 2. Materials and Methods

### 2.1. Materials

The walnut peptide KG-7, identified from a walnut protein hydrolysate fraction with a molecular weight <3 kDa after enzymatic and ultrafiltration, was determined using HPLC-MS/MS. KG-7 was synthesized (98% purity) by Jiangsu Jitai Peptide Technology Co., Ltd. (Yancheng, China) and used for subsequent experiments. Titanium dioxide and scopolamine were obtained from Aladdin Biochemical Technology Co., Ltd. (Shanghai, China). AChE and ACh kits were purchased from the Nanjing Jiancheng Bioengineering Institute (Nanjing, China). Antibodies against ZO-1 (1:2000), Occludin (1:1000), Claudin-2 (1:1000), BCRP (1:2000), LRP1 (1:5000), Hes1 (1:2000), and P-glycoprotein (P-gp) (1:2000) were purchased from ABclonal Technology (Wuhan, China). Radioimmunoprecipitation (RIPA) lysate and the ultra-sensitive ECL chemiluminescence kit were purchased from Beyotime Biotechnology Co., Ltd. (Shanghai, China). The HE staining kit was purchased from Solarbio Science & Technology Co., Ltd. (Beijing, China). Neutral gel was purchased from Sinopharm Chemical Reagent Co., Ltd. (Shanghai, China), and the 4% paraformaldehyde (PFA) fixation solution was purchased from Dalian Meilun Biotechnology Co., Ltd. (Dalian, China). All other chemicals and reagents used in this study were of analytical grade.

### 2.2. Animal Treatment

Male C57BL/6J mice (age 6–8 weeks, body weight 20 ± 2 g) were obtained from Liaoning Changsheng Biotechnology Co., Ltd. (Certificate No.: SXCK (Liao) 2022-0012, Shenyang, China). Animals were housed under specific pathogen-free conditions with controlled temperature (22 ± 2 °C), humidity (40–60%), and a 12/12 h light–dark cycle. They had free access to food and water and were given a 1-week acclimation period. Following 7-day acclimatization, 50 mice were equally and randomly assigned to five groups (*n* = 10 per group): control, model, KG-7-L (40 mg/kg KG-7 treatment), KG-7-M (80 mg/kg KG-7 treatment), and KG-7-H (120 mg/kg KG-7 treatment). Daily monitoring of physiological parameters (body mass and food and water consumption) was conducted at 08:00 throughout the pre-experimental phase to ensure metabolic equivalence among groups before behavioral testing. All experimental procedures were conducted in strict conformity with laboratory animal care and use protocols and were approved by the Laboratory Animal Welfare and Ethics Committee of Jilin Agricultural University (Animal Use License No. 2025 05 15 005).

### 2.3. Drug Administration for Animals

**Figure 1:**
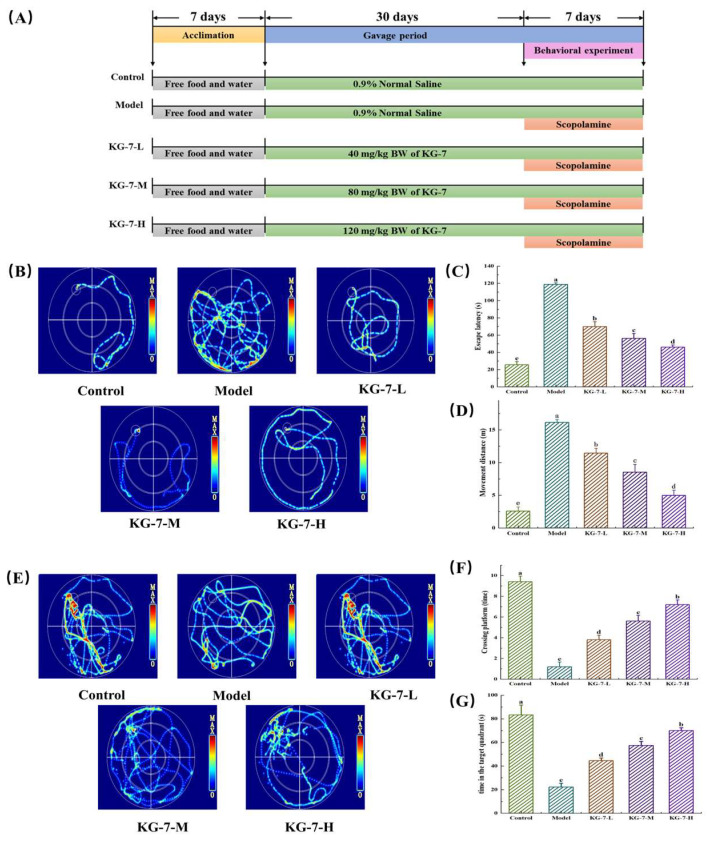
Effects of KG-7 on learning and memory deficits in scopolamine-induced mice observed within the Morris Water Maze. (**A**) Animal experimental protocol. (**B**) Trajectory maps of the place navigation trial. (**C**) Escape latency. (**D**) Distance of movement. (**E**) Trajectory maps of the spatial probe trial. (**F**) Number of platform crossings. (**G**) Target quadrant dwell time. Data are presented as mean ± SD (n = 10). Different letters represent statistically significant differences at the *p* < 0.05 level.

As shown in Figure 1A and Table 1, following acclimatization, mice in the KG-7-L, KG-7-M, and KG-7-H groups received daily oral administration of the designated KG-7 doses via gastric intubation for 30 days, whereas the control and model groups were administered equivalent volumes of 0.9% saline. Subsequently, the experimental groups were intraperitoneally administered 1 mg/kg scopolamine hydrobromide dissolved in 0.9% saline for 7 days as described by Geng et al. [10], while the control group received intraperitoneal saline injections for the same duration. At the end of the experiment, the mice were euthanized by cervical dislocation, followed by rapid brain dissection and snap-freezing in liquid nitrogen for biochemical analyses.

### 2.4. Morris Water Maze Test

The MWM protocol was implemented as described by Geng et al. [10] with modifications, using a circular tank (diameter: 120 cm, height: 60 cm) filled to a depth of 30 cm with temperature-controlled water (22–25 °C). The platform was placed 1 cm underwater. The aqueous environment was rendered opaque using titanium dioxide suspension to eliminate visual cues, with continuous monitoring to maintain the water at 22–25 °C. The mice were placed at the entry point facing the pool wall. Swimming trajectories were recorded using a computerized video-tracking system, including escape latency (≤120 s), path distance, time spent in the target quadrant, and the number of platform crossings.

### 2.5. Analysis of AChE Activity and Ach Level in the Hippocampus

Hippocampal tissues were isolated within 30 min of sacrifice and stored at −80 °C until the start of the experiment. ACh levels and AChE activity were determined using ACh level and AChE activity assay kits, respectively. The hippocampus was homogenized with saline at a ratio of 1:9 on ice and centrifuged at 3000× *g* for 10 min at 4 °C for the determination of AChE activity and ACh level. The supernatant was collected for measurements. The protein content was determined using a BCA kit, AChE activity and ACh levels were determined using kits from the Nanjing Jiancheng Bioengineering Institute(Nanjing, China).

### 2.6. HE Staining of Hippocampal Tissue

HE staining of hippocampal sections was performed according to Han et al. [18] with protocol modifications. Following rapid dissection, brain specimens were immersion-fixed in 4% PFA for 24 h at 22 ± 2 °C, processed through a graded ethanol dehydration series, embedded in paraffin, and sectioned at 5 μm using a rotary microtome. Deparaffinization was carried out in xylene, followed by rehydration through a descending ethanol gradient. The sections were counterstained with HE, dehydrated using an ascending ethanol series, cleared in xylene, and mounted with neutral resin. Images were then captured using an imaging system for analysis.

### 2.7. Immunohistochemical Staining of Hes1 Protein in the Hippocampus

Staining was performed according to the protocol described by Yan et al. [19] with slight modifications. Tissue sections were deparaffinized in xylene, rehydrated using a graded ethanol series, and immersed in distilled water. After rehydration, the mouse hippocampal tissue was placed in a repair cassette containing 3% bovine serum albumin (BSA) to uniformly cover the tissue and incubated at 25 °C. Sections were then incubated with rabbit anti-Hes1 primary antibody overnight at 4 °C, followed by incubation with HRP-conjugated goat anti-rabbit IgG at 25 °C for 1 h. Chromogenic visualization was performed using a 5 min diaminobenzidine (DAB) substrate incubation, followed by HE counterstaining, dehydration through graded ethanol, xylene clearing, and mounting with neutral resin. Images were captured using an imaging system for analysis.

### 2.8. Fluorescence Imaging After Oral Administration

According to the method described by Li et al. [16], mice were orally administered B-KG-7 and rhodamine B (80 mg/kg BW) and fasted for 24 h before the experiment. Mice were sacrificed at 0, 1, 2, 4, 8, 12, and 24 h, and tissues (brains and livers) were collected. Fluorescence imaging was then conducted using an IVIS Lumina XR system.

### 2.9. Immunohistochemical Staining of P-gp Protein in the Small Intestine

Immunohistochemical staining was performed as described by Li et al. [20] with slight modifications. Small intestinal tissue specimens were fixed in 4% PFA, processed through a graded ethanol dehydration series, and embedded in paraffin. The samples were sectioned, and nonspecific binding was blocked with 3% BSA for 1 h at room temperature. Sections were incubated with rabbit anti-P-gp antibody overnight at 4 °C, followed by HRP-conjugated goat anti-rabbit IgG incubation at 25 °C for 1 h. Chromogenic visualization was achieved through 5 min DAB substrate incubation, followed by HE counterstaining, dehydration in ascending ethanol gradients, xylene clearing, and mounting with neutral resin.

### 2.10. Immunofluorescence Staining Analysis of ZO-1 and Occludin

The procedure described by Burgueño et al. was slightly modified [21]. Sectioning was performed using the same procedure as the immunohistochemical sections. Sections were deparaffinized and rehydrated by adding an appropriate amount of treatment solution. They were then incubated with primary antibody overnight at 4 °C, followed by secondary antibody incubation at 25 °C for 1 h. 2-(4-Amidinophenyl)-6-indolecarbamidine dihydrochloride (DAPI) was applied and incubated for 10 min at room temperature for nuclear staining.

### 2.11. Western Blot Analysis of the KG-7 Absorption Pathway

Western blotting was performed as previously described with slight modifications [22]. Small intestinal tissues were lysed at a 99:1 ratio using RIPA lysis buffer (Beyotime Biotechnology Co., Ltd. (Shanghai, China)) containing phenylmethylsulfonyl fluoride (PMSF). The homogenate was centrifuged at 4 °C (10,000× *g* for 10 min), and the supernatant was collected. Proteins were separated by electrophoresis on 8–12% sodium dodecyl sulfate–polyacrylamide gels and transferred to polyvinylidene fluoride (PVDF) membranes. The membranes were blocked for 1 h, washed three times, and incubated overnight at 4 °C with primary antibodies ZO-1, Occludin, Claudin-2, BCRP, P-gp, and LRP1. The membranes were then incubated with secondary antibodies for 1 h at room temperature and washed three times. The electrochemiluminescent substrate was finally examined using an ImageQuant LAS 500 system.

### 2.12. Blinding

To minimize potential bias in both experimental operation and result evaluation, a strict blinding protocol was implemented. Specifically, all samples were coded and randomly re-ordered prior to analysis. Researchers in the outcome assessment and data analysis stages remained completely unaware of group identities, receiving only coded samples.

### 2.13. Statistical Analysis

Each experiment was replicated at least three times in parallel, and the data were expressed as means ± standard deviations (SD). Data were analyzed using SPSS 19.0 software (SPSS Inc., Chicago, IL, USA). One-way analysis of variance followed by the Tukey HSD test was performed to compare the differences between groups, and the significance level was set at *p* < 0.05.

## 3. Results and Discussion

### 3.1. KG-7 Improved the Behavioral Performance of Scopolamine-Induced Mice in the MWM

We evaluated whether KG-7 ameliorates scopolamine-induced memory deficits by recording the behavioral performance of mice within the MWM. As shown in Figure 1, during the place navigation trial, the model group exhibited disorganized swimming patterns and purposeless searches behavior, in contrast to the control group. Compared to the model group, the movement trajectories of the KG-7-L, KG-7-M, and KG-7-H groups were more organized and goal-directed. Latency and moving distance were significantly and dose-dependently lower in the KG-7-treated groups than in the model group (*p* < 0.05). In the spatial probe trial, the model group displayed disorganized searches behavior and failed to retain platform localization memory compared to the control group. In contrast, the KG-7-L, KG-7-M, and KG-7-H groups exhibited more purposeful movement trajectories. Significant dose-dependent enhancements were observed in the number of platform crossings, and the target quadrant dwell time was significantly increased (*p* < 0.05). Fu et al. [23] demonstrated that the co-administration of WNP and GSE improved scopolamine-induced memory deficits in MWM tests. Mechanistic analyses showed that memory improvement was mainly accomplished by protecting the integrity of neurons and modulating cholinergic neurotransmission. Similarly, Zheng et al. [24] reported that SSDAFFPFR and SNVFDMF enhanced MWM performance in scopolamine-treated mice. Collectively, the MWM test results indicate that KG-7 significantly improved scopolamine-induced memory deficits and the overall behavioral performance, demonstrating its ameliorative effects on scopolamine-induced memory impairments.

### 3.2. KG-7 Ameliorated Hippocampal Neurotransmitters Disorders in Mice

**Figure 2:**
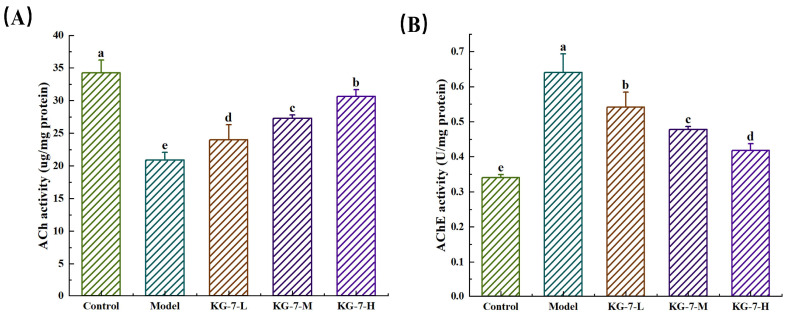
The protective effect of KG-7 on scopolamine-induced memory deficits by measuring changes in AChE activity and ACh levels. (**A**) Effect of KG-7 on ACh levels. (**B**) Effect of KG-7 on AChE activity. Data are presented as mean ± SD (n = 10). Different letters represent statistically significant differences at the *p* < 0.05 level.

This study aimed to elucidate the protective effect of KG-7 on scopolamine-induced memory deficits by measuring changes in AChE activity and ACh levels. As shown in Figure 2, compared to the control group, AChE activity in the hippocampus of the model group was significantly increased, whereas ACh levels were significantly decreased (*p* < 0.05). KG-7 treatment reversed these alterations in a dose-dependent manner, significantly decreasing AChE activity and increasing ACh levels (*p* < 0.05). ACh is the principal neurotransmitter that mediates hippocampal memory consolidation [25], and decreased ACh levels are associated with memory deficits [26]. AChE catalyzes ACh hydrolysis at central synapses, making it a primary pharmacological target for improving memory deficits [27]. The experimental data demonstrated that KG-7 reduced AChE activity and increased ACh levels. Combined with the MWM tests, these findings further confirm that KG-7 improves scopolamine-induced memory deficits. The inhibitory effects on AChE may be related to its amino acid composition [28]. Basic amino acids can modulate AChE activity by forming stable complexes with peripheral anionic sites [29], and this modulation may occur via competitive inhibition that reduces ACh hydrolysis. Structural studies also suggest that glycosylation of lysine residues in AChE alters the conformation of catalytic site residues (Trp-86 and His-447), ultimately hindering ACh hydrolysis [30]. Zhao et al. [30] reported the cognition-enhancing efficacy of natto-derived Gln-Gln via cholinergic potentiation. Equivalent doses of free Gln failed to reproduce these effects, highlighting peptide-specific bioactivity and indicating that a combination of amino acids can be more effective than individual amino acids. Therefore, the amino acid combination in KG-7 may contribute to its ability to modulate AChE activity and Ach levels. Through behavioral tests, Zhao et al. [31] further showed that the sea cucumber peptide FYDWPK improves scopolamine-induced memory deficits. This peptide shares structural features with KG-7, including basic amino acid residues associated with AChE inhibitory activity. In conclusion, the amino acid combination in KG-7 contains the basic amino acids lysine and histidine, which are likely responsible for regulating ACh levels and AChE activity. These results show that KG-7 reduced AChE activity and increased ACh levels in mice with scopolamine-induced memory deficits, thereby improving memory.

### 3.3. KG-7 Ameliorates Neuronal Cell Damage in Hippocampal Subfields

**Figure 3:**
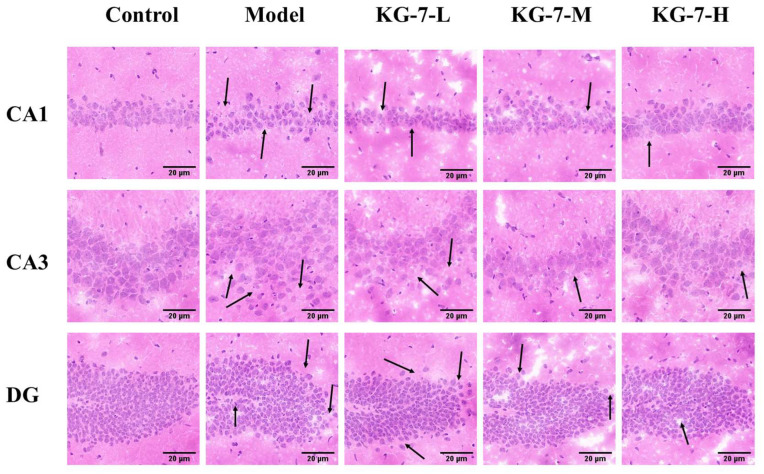
The protective effect of KG-7 on hippocampal neurons was analyzed using HE staining. The presence of pathological damage is marked by arrows. Scale bar = 20 μm.

The protective effect of KG-7 on hippocampal neurons was analyzed using HE staining. As shown in Figure 3, compared with the control group, the cells in the CA1 region of the model group were arranged irregularly, reticularly separated, and pyknotic. Compared with the model group, the structure of CA1 cells in the KG-7-L, KG-7-M, and KG-7-H groups was clearer; however, a small number of local pyramidal cells still showed blurred outlines and pyknosis, although the overall damage was alleviated. In the model group, morphological changes in the CA3 region were obvious; the number of nerve cells decreased, and the pyramidal cells contracted and were arranged disorderly. Compared to the model group, the degree of cell shrinkage, cell damage, and CA3 region impairment in the KG-7-L, KG-7-M, and KG-7-H groups was reduced. The model group also showed obvious morphological changes in the dentate gyrus (DG) region, with a reduced number of nerve cells and irregular morphology. Compared with the model group, the neurons in the DG region in the KG-7-L, KG-7-M, and KG-7-H groups were clearer and more intact, and the damage was alleviated. The hippocampus serves as a critical hub for spatial information processing and explicit memory consolidation, primarily mediated by CA1 pyramidal neurons as its principal efferent pathway [32]. The CA3 region of the hippocampus serves as a key center for both encoding and retrieving memory [33]. Additionally, the DG plays a dual role in adult neurogenesis and episodic memory discrimination [34]. KG-7 administration attenuated neuronal damage across the hippocampal subfields (CA1, CA3, and DG), concomitantly enhancing neurogenesis and improving spatial memory encoding capacity. Using hippocampal HE staining, Shen et al. [35] established the neuroprotective effects of the walnut peptide YVPFPLP on scopolamine-induced CA1 neuronal damage. The golden mushroom-hydrolyzing peptide YVYAETY ameliorated scopolamine-induced memory deficits by reducing hippocampal neuronal damage [36]. The walnut peptide LPLLR reduced neuronal damage in the hippocampal CA1 subfield and protected against memory deficits in a mouse model of dextran sodium sulfate–induced colitis [37]. These bioactive peptides share common structural features, including a low molecular weight and increased hydrophobicity. Low-molecular-weight peptides demonstrate enhanced neuroprotective efficacy against memory decline [38]. Peptides with high hydrophobicity exert beneficial neuroprotective effects because of their greater ability to penetrate the cell membrane and reach target sites [39]. The presence of hydrophobic amino acids Leu, Phe, and Pro in our KG-7 further confirmed its ability to cross the blood–brain barrier and exert its activity. Zheng et al. showed that natto Gln-Leu, Gln-Ile, Gln-Gln, and Ala-Tyr significantly attenuated scopolamine-induced memory impairment and increased ACh levels; however, equimolar amounts of Gln as a free amino acid did not show significant effects [11]. This suggests that the amino acid composition also contributes to the memory-improving activity of the peptide. In conclusion, the structural characteristics and amino acid composition of KG-7, particularly its low molecular weight and hydrophobic properties, collectively contribute to the observed neuroprotective effects in the hippocampal subfields, ultimately alleviating memory and learning deficits.

### 3.4. KG-7 Regulated Hes1 Protein Expression in the Hippocampus

**Figure 4:**
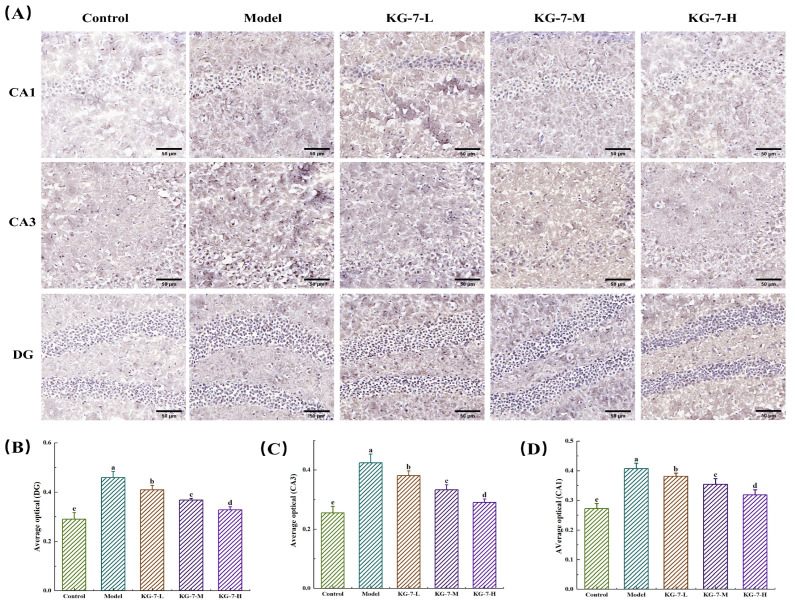
The effect of KG-7 on Hes1 protein expression was evaluated using immunohistochemical staining. (**A**) Representative photomicrographs of immunohistochemical staining for Hes1 among different groups. Scale bar = 50 μm. (**B**) Mean optical density in the CA1 region. (**C**) Mean optical density in the CA3 region. (**D**) mean optical density in the DG region. Data are presented as mean ± SD (n = 10). Different letters represent statistically significant differences at the *p* < 0.05 level.

Hes1 plays a pivotal role in regulating the cell cycle, proliferation, differentiation, survival, and neuronal apoptosis [40]. The Hes1 protein expression was revealed through immunohistochemical analysis. Protein localization was identified by brown-yellow chromogenic particles, with particle density positively correlated with antigen concentration. As shown in Figure 4, Hes1 expression was significantly upregulated in the hippocampal CA1, CA3, and DG regions of the model group compared to the control group (*p* < 0.05). Compared to the model group, the KG-7-L, KG-7-M, and KG-7-H groups showed significant reductions in Hes1 protein expression in the CA1, CA3, and DG subfields (*p* < 0.05). Hes1 protein expression in the CA1, CA3, and DG subfields of the KG-7-H group was significantly lower than that in the KG-7-L and KG-7-M groups (*p* < 0.05). Hes1 regulates neural stem cell survival and proliferation, and HE staining revealed that KG-7 ameliorated hippocampal neuronal damage and increased neuronal density. These findings suggest that KG-7 promotes neuronal proliferation via the modulation of Hes1 expression. Yan et al. [19] reported that the shrimp peptide QMDDQ downregulates hippocampal Hes1 expression and improves memory, which is consistent with our KG-7 findings. Du et al. [41] demonstrated that sodium fluoride impairs neurogenesis via Notch1 activation, whereas carvacrol mitigates neuronal damage through Hes1 inhibition and the promotion of neurogenesis. As Hes1 is a key Notch signaling effector, KG-7 may exert its effects through Notch1-mediated regulation of Hes1 expression. In conclusion, the pathological upregulation of Hes1 in animal models confirmed successful memory deficit induction and identified Hes1 as a critical therapeutic target. Through Hes1 downregulation, KG-7 orchestrates neuronal proliferation and damage repair mechanisms, ultimately ameliorating scopolamine-induced memory deficits.

### 3.5. Ex Vivo Fluorescence Imaging of the Brain After Oral Administration of KG-7

**Figure 5:**
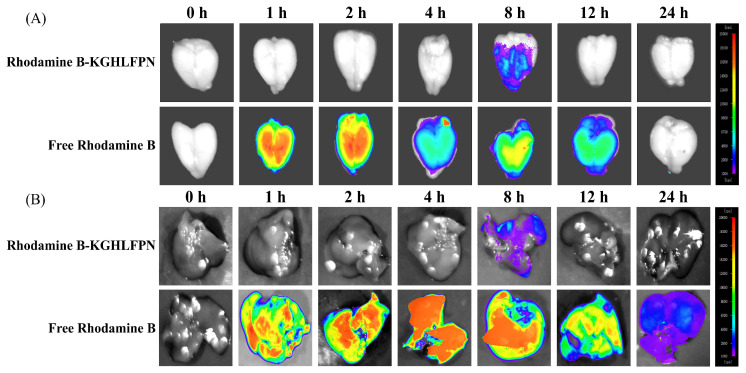
Ex vivo fluorescence imaging of the brain and liver in mice at 1, 2, 4, 8, 12, and 24 h after oral administration of rhodamine B-KG-7 and rhodamine B (80 mg/kg BW). (**A**) In vitro fluorescence imaging of the brain. (**B**) In vitro fluorescence imaging of the liver.

Ex vivo fluorescence imaging of isolated organs was used to track the biodistribution of rhodamine B-KG-7 in the mouse brain over 0–24 h. As shown in Figure 5A, rhodamine B monomer controls confirmed that the rhodamine B-KG-7 fluorescence intensity reflected KG-7 metabolic processing in vivo. Ex vivo imaging of brains harvested from mice at different time points after oral administration of B-KG-7 revealed that the fluorescence intensity peaked at 8 h, with complete signal disappearance after 12 h, suggesting complete cerebral metabolism of KG-7 within this timeframe. An obvious B-KG-7 fluorescence signal was observed in the brain at 8 h, indicating that KG-7 entered the brain through the blood. Cerebral fluorescence showed progressive attenuation after 8 h and reached complete clearance by 12 h. Therefore, 8 h was determined as the optimal time for oral administration of B-KG-7 to exert its effect in the brain. As shown in Figure 5B, a fluorescent signal was detected in the livers harvested 8 h after oral administration of B-KG-7 and disappeared at 12 h, consistent with the changes in the brain. Li et al. [8] showed that KG-7 completed small intestinal absorption within 4 h and that small intestinal absorption reached saturation at 8 h. However, our results showed that fluorescence in the brain reached a maximum at 8 h, indicating that KG-7 reached the brain through blood transport at 4–8 h. Yang et al. [42] reported detectable FITC-FPF in brain tissue within 1 h post-gavage (40 mg/kg BW) using isolated organ fluorescence imaging. Fluorescence imaging by Min et al. [43] showed that fluorescence appeared in mouse brains at 2–12 h, indicating that the walnut peptide TW-7 entered the brain at 2 h. Gu et al. [44] developed the Augur model, a method for identifying peptides that can enter the brain based on the physicochemical properties of amino acids, and found that the peptides present several characteristics, such as glycine, lysine, leucine, and proline. Since KG-7 is structurally composed of glycine, lysine, leucine, and proline, it is likely that KG-7 can enter the brain because of its amino acid composition. In conclusion, the KG-7 demonstrated peak brain and liver accumulation at 8 h post administration, establishing a pharmacokinetic basis for brain-targeted therapeutic development.

### 3.6. Effect of KG-7 on P-gp Protein Expression in the Small Intestine

**Figure 6:**
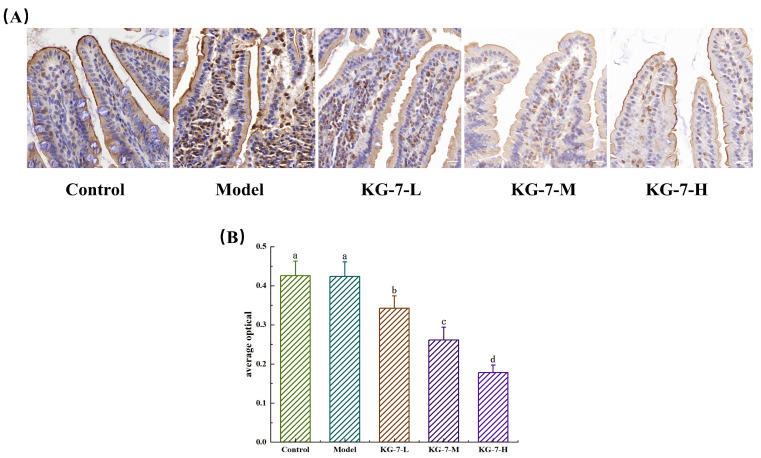
The effect of KG-7 on P-gp protein expression was evaluated through immunohistochemical staining in the small intestine. (**A**) Immunohistochemical images. Scale bar = 20 μm. (**B**) Quantitative analysis of average optical density. Data are presented as mean ± SD (n = 10). Different letters represent statistically significant differences at the *p* < 0.05 level.

As a key efflux transporter, P-gp critically modulates oral drug absorption and tissue distribution [45]. Therefore, in the present study, the effect of the walnut peptide KG-7 on the efflux protein P-gp was investigated through the immunohistochemical analysis of P-gp expression in the small intestine. As shown in Figure 6A, the immunohistochemical results revealed brownish-yellow particulate staining, indicating P-gp immunoreactivity. No statistically significant intergroup differences (*p* > 0.05) were observed in P-gp protein expression levels between the control and model groups. KG-7 administration dose-dependently attenuated P-gp protein expression compared to the model group, with a progressive reduction observed with increasing dosage regimens. As shown in Figure 6B, there was no significant difference (*p* > 0.05) between the mean optical density values of the control group (0.45 ± 0.84) and the model group (0.42 ± 0.59). P-gp critically regulates the transmucosal transport of natural active ingredients, thereby limiting their systemic absorption [46]. The inhibition of P-gp improves intestinal absorption and distribution in tissues [47]. This P-gp suppression mechanism potentially enhances the autologous absorption efficiency of KG-7. Quercetin potentiates AR-C17 bioavailability via P-gp inhibition, thereby facilitating transmembrane transport [48]. The combined regimen of tanshinones with Danxingfang augmented cryptotanshinone absorption through P-gp inhibition, significantly elevating its oral bioavailability [49]. Mandal et al. [50] engineered a His-Leu-LPV prodrug that exhibited reduced affinity for P-gp and MRP2 efflux transporters, thereby enhancing the bioavailability of lopinavir through attenuated exocytosis. By suppressing P-gp expression, KG-7 may concurrently enhance the activity of P-gp substrate drugs, thereby mitigating efflux-mediated clearance. In conclusion, the walnut peptide KG-7 demonstrated P-gp efflux inhibitory activity, potentially enhancing its autologous absorption, while improving the bioavailability of P-gp substrates through transporter modulation.

### 3.7. Effect of KG-7 on Distribution of TJs in the Small Intestine

**Figure 7:**
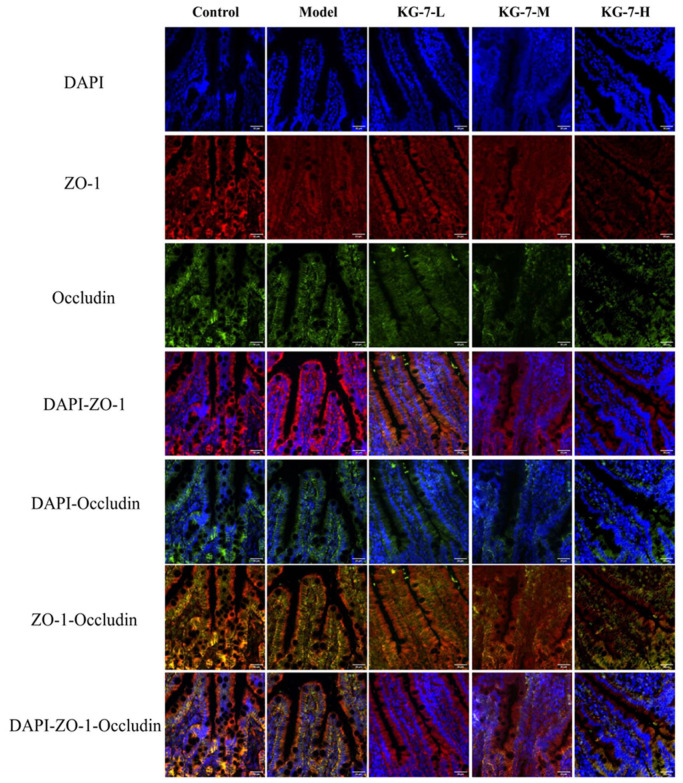
Immunofluorescence staining of TJ proteins (ZO-1 and Occludin) in mouse intestinal villi. Scale bar = 20 μm.

Immunofluorescence staining was used to assess the distribution of Occludin and ZO-1 in intestinal villi. As shown in Figure 7, the control group exhibited a continuous network of Occludin and ZO-1 between adjacent cells, with the nucleus enclosed by this structure. The model group showed a distribution pattern similar to that of the control group; however, fluorescence intensity was not significantly altered. In contrast, the KG-7-treated groups displayed dose-dependent reductions in the fluorescence intensity of both Occludin and ZO-1, accompanied by decreased reticular structural integrity compared to the control group. The KG-7-H group showed the lowest fluorescence intensity and the most pronounced disruption of the reticular network. TJs form intercellular adhesion complexes that regulate paracellular permeability across epithelial and endothelial barriers [51]. Their dynamic nature is controlled by multiple intracellular signaling pathways that influence TJ expression and localization [52]. KG-7 downregulated TJ expression and enhanced paracellular permeability, indicating that TJ-mediated paracellular transport is the predominant absorption pathway. Li et al. [16] similarly observed that QMDDQ induced TJ alterations in Caco-2 monolayers, characterized by reduced Occludin and ZO-1 fluorescence and increased paracellular flux. These findings confirmed that TJs mediate paracellular absorption, with QMDDQ primarily relying on this pathway. Glycyrrhizin (GL) interacts with the PDZ1 domain of ZO-1 via ionic coordination between its glucuronic acid carboxyl group (-COOH) and the carboxylate-binding loop of ZO-1. These molecular interactions modulate TJ integrity, thereby enhancing GL absorption [53]. Natural ligands of the PDZ domains depend on a C-terminal carboxylate group to bind to the PDZ1 carboxylate-binding loop. KG-7 is structurally similar to GL in that regard, as its C-terminus contains an aspartic acid residue whose side chain provides carboxyl groups. Therefore, the KG-7 likely binds to the first PDZ domain of ZO-1, facilitating TJ-mediated paracellular transport and enhancing intestinal epithelial permeability.

### 3.8. Effect of KG-7 on Small Intestinal Tight Junction and Efflux Proteins

**Figure 8:**
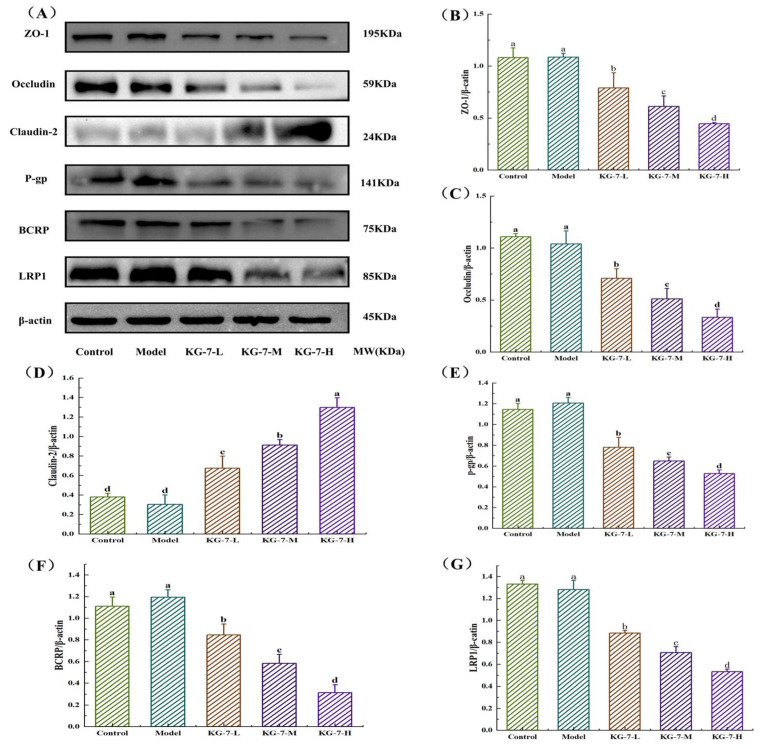
The paracellular absorption pathway of KG-7 was demonstrated through protein immunoblot analysis. (**A**) Representative western blot bands. (**B**) ZO-1 expression. (**C**) Occludin expression. (**D**) Claudin-2 expression. (**E**) P-gp expression. (**F**) BCRP expression. (**G**) LRP1 expression. Data are presented as mean ± SD. Different letters represent statistically significant differences at the *p* < 0.05 level.

The present study further analyzed the expression of small intestinal TJs and efflux proteins using western blotting. As shown in Figure 8, western blot analysis revealed no significant changes in the expression levels of ZO-1, Occludin, Claudin-2, BCRP, P-gp, or LRP1 in the model group compared to the control group (*p* > 0.05). In contrast, KG-7 treatment induced a dose-dependent downregulation of ZO-1, Occludin, BCRP, P-gp, and LRP1, while upregulating Claudin-2 expression across all dosage groups (*p* < 0.05). Immunofluorescence analysis showed that modulation of ZO-1 and Occludin expression was consistent with enhanced paracellular transport in the intestinal epithelium. Furthermore, Claudin-2 is expressed in TJs of intestinal epithelial cells and is closely associated with paracellular permeability [54]. Wang et al. [55] demonstrated that IL-22 upregulated Claudin-2 expression via the JAK/STAT pathway, resulting in increased paracellular permeability. Consistent with these findings, KG-7 elevated Claudin-2 levels, reinforcing paracellular transport. Using small intestinal immunohistochemistry, we investigated the effect of KG-7 on the efflux transporter, P-gp. In this study, we also investigated BCRP, an efflux transporter expressed on the apical membrane of small intestinal epithelial cells that limits absorption through its efflux mechanism [56]. Liu et al. [57] showed that genetic ablation of BCRP and MRP2 in mice blocked the efflux of wogonin, resulting in increased plasma wogonin levels and enhanced bioavailability and pharmacological activity. In addition, we examined the expression of LRP1, an endocytic and cell-signaling transmembrane protein. LRP1 plays a key role in regulating the paracellular pathway through which blood proteins enter the brain, and its ablation leads to TJ degradation and reduced P-gp expression [58]. KG-7 inhibited LRP1 expression; therefore, it may regulate TJs and P-gp through LRP1-mediated mechanisms. In conclusion, KG-7 decreased the expression of ZO-1 and Occludin while increasing Claudin-2 levels, verifying its entry into the blood circulation through paracellular absorption. KG-7 also inhibited the expression of efflux proteins (BCRP and P-gp) and LRP1, thereby promoting the absorption of active ingredients.

## 4. Conclusions

In summary, our findings demonstrate that KG-7 ameliorates scopolamine-induced memory deficits and is absorbed in the small intestine via the paracellular pathway. KG-7 reduced the overexpression of Hes1 in the hippocampus and attenuated pathological changes in the CA1, CA3, and DG subfields, thereby improving the behavioral performance of memory-impaired mice in the MWM tests. Additionally, KG-7 reduced AChE activity and increased ACh levels, further validating its functional role in improving memory. During absorption, the expression of ZO-1 and Occludin in the small intestine was reduced, whereas Claudin-2 expression was increased, enabling paracellular entry into the bloodstream. KG-7 then entered the brain via blood transport. In addition, KG-7 reduced the expression of efflux proteins (BCRP and P-gp) and LRP1, suggesting improved bioavailability of KG-7 in vivo and potential regulatory effects on TJ mechanisms. In conclusion, the results demonstrate that KG-7 has memory-enhancing activity and absorption characteristics in vivo; as such, it has potential for use as a bioactive ingredient in memory-functional foods. This study provides a theoretical reference for understanding the in vivo transport mechanisms of the small peptides.

## Figures and Tables

**Table 1:** Group and dose of mice.

Group	Acclimatization 7 Days	Gavage 30 Days		Model and Behavior Test 7 Days	
		Sample	Concentration	Sample	Concentration
Control	–	Normal Saline	0.9%	Normal Saline	0.9%
Model	–	Normal Saline	0.9%	Scopolamine	1 mg/kg
KG-7-L	–	KG-7	40 mg/kg	KG-7 + Scopolamine	40 mg/kg + 1 mg/kg
KG-7-M	–	KG-7	80 mg/kg	KG-7 + Scopolamine	80 mg/kg + 1 mg/kg
KG-7-H	–	KG-7	120 mg/kg	KG-7 + Scopolamine	120 mg/kg + 1 mg/kg

## Data Availability

The original contributions presented in this study are included in the article. Further inquiries can be directed to the corresponding author..

## Abbreviations

Lys-Gly-His-Leu-Phe-Pro-Asp (KG-7), Alzheimer’s disease (AD), acetylcholinesterase (AChE), hematoxylin and eosin (HE), tight junction (TJ), Gln-Met-Asp-Asp-Gln (QMDDQ), Morris water maze (MWM), acetylcholine (ACh), P-glycoprotein (P-gp), radioimmunoprecipitation (RIPA), paraformaldehyde (PFA), bovine serum albumin (BSA), diaminobenzidine (DAB), 2-(4-Amidinophenyl)-6-indolecarbamidine dihydrochloride (DAPI), phenylmethylsulfonyl fluoride (PMSF), polyvinylidene fluoride (PVDF), standard deviations (SD), dentate gyrus (DG), glycyrrhizin (GL).
